# A study of forensic genetics: ITO index distribution and kinship judgment between two individuals

**DOI:** 10.1515/biol-2022-0786

**Published:** 2023-12-28

**Authors:** Huanchang Jiang, Qinglian Jiang, Shuzhen Wu

**Affiliations:** The First Clinical Medical College of Wenzhou Medical University, Wenzhou, Zhejiang Province, 325035, China; Wenzhou Tianzheng Judicial Expertise Office, Wenzhou, Zhejiang Province, 325000, China; Judicial Expertise Center of Wenzhou Medical University, No. 268, Xueyuan West Road, Lucheng District, Wenzhou City, 325000, Zhejiang Province, China

**Keywords:** forensic genetics, short tandem repeat, ITO index, kinship testing, homologous genes

## Abstract

According to Mendel’s law of genetic separation, there must be a certain blood relationship between members of the same family, and two individuals with blood relations must have the following three situations, that is, there are two homologous genes (I denotes), one homologous gene (T denotes), and no homologous genes (O denotes), which is the ITO index for calculating the blood relationship between two individuals. The AGGU Expressmarker 22 kit, ABI 3500 genetic analyzer, and GeneMapper ID-X v1.5 software were used to statistically analyze the ITO index of the gene locus of 5 kinds of samples, 28 pairs of monozygotic twins (MT), 4,000 pairs of parent–offspring (PO), 392 pairs of full sibling (FS), 138 pairs of half-siblings (HS) (including grandchildren, uncles, and nephews) and 3,500 pairs of unrelated individuals (UI). Observing the median distribution of ITO index found that from MT, PO, FS, HS to UI, the more distant the kinship, the smaller the ITO index. Full sibling index (FSI)/half-sibling index (HSI) ≥ 1 can be used as the FS discriminant standard, FSI/HSI < 1 can be used as the HS discriminant standard. According to the distance of kinship, from the direction of MT, PO, FS, HS, and UI, the proportion of the maximum ITO index of the same type of sample in the true kinship index item showed a decreasing trend. ITO index is an important statistical means to identify the kinship between two individuals, according to which the ITO index can accurately determine the kinship between individuals, which has high application value. MT index is not 0 to identify relatives as MT, PO index is an important indicator to distinguish between relatives as PO and FS. The critical values of ITO index discriminant values for UI and HS need to be further studied.

## 1 Introduction

There are 0, 1, and 2 identical-by-descent alleles per locus between relatives (probability 0.25, 0.5, and 0.25, respectively), which is the basic principle of full sibling (FS) identification as another type of major kinship identification in addition to paternal identification [1,2]. Based on this principle, the autosomal short tandem repeat (STR) has become a widely used genetic marker in the field of forensic physical identification [3,4]. Li and Sacks first proposed the random ITO transform matrix method in 1954 for obtaining joint STR genotype distribution and genotypic correlation between any particular non-breeding relative pair [5]. According to Mendelian hereditary laws, there must be a certain blood relationship between members of the same family. In the ITO calculation method, I, T, and O represent the probability of individual 2 developing the genotype when two individuals in a pair of samples have 2, 1, or no homologous genes [6]. At present, the application of ITO methods has been reported in the identification of FS and half-siblings (HS), and occasionally in the identification of cousins, and the survey statistics of ITO index distribution of other sample types have not been reported [7]. At present, the commonly used detection system for paternity testing is AGCU EX22, and the 21 autosomal STR loci observed in this system are D3S1358, D13S317, D7S820, D16S539, Penta E, D2S441, TPOX, TH01, D2S1338, CSF1PO, Penta D, D10S1248, D19S433, vWA, D21S11, D18S51, D6S1043, D8S1179, D5S818, D12S391, and FGA locus [8]. In this article, the ITO method is used to calculate the ITO index of autosomal STR typing results of different related individuals, and the distribution range of ITO index of the same type of samples is counted to understand its distribution law, which provides a theoretical basis for the application of ITO index to determine kinship.

## 2 Materials and methods

### 2.1 Samples

The samples were collected from the daily examination accumulation of the Judicial Expertise Center of Wenzhou Medical University in 2013–2022, the sample information was collected with informed consent. It was approved by the Ethics Committee of the Forensic Appraisal Center of Wenzhou Medical University (WYDSJZX2023-1), and all samples obtained oral informed consent of the samples. The sample included 28 pairs of monozygotic twins (MT), 4,000 pairs of parent–offspring (PO), 392 pairs of FS, 138 pairs of HS (including grandchildren, uncles, and nephews), and 3,500 pairs of unrelated individuals (UI), one pair representing two individuals. All MT and FS samples were from offspring of triplet paternity testing families whose parents were also involved and supported kinship; 48 pairs of HS samples were from offspring individuals of half-sitomy or half-father triad families; the UI samples were from the triad samples from the parents of the test (excluding the parents’ combination with the same surname). In all FS samples and HS samples, the transmission of autosomal STR locus alleles that support kinship conforms to Mendelian inheritance laws and there are no mutations.


**Informed consent:** Informed consent has been obtained from all individuals included in this study.
**Ethical approval:** The research related to human use has been complied with all the relevant national regulations, institutional policies, and in accordance with the tenets of the Helsinki Declaration, and has been approved by the Ethics Committee of the Forensic Appraisal Center of Wenzhou Medical University (WYDSJZX2023-1).

### 2.2 Instruments and reagents

A 96-well Veriti PCR amplification instrument, ABI 3500 genetic analyzer (AB Company, USA), AGCU EX22, AGCU 21 + 1, AGCU X19 and AGCU Database Y24 kits (Wuxi Zhongde Meilian Biotechnology Co., Ltd), and GeneMapper ID-X 1.5 software for genotype analysis were used.

### 2.3 DNA extraction and autosomal STR loci detection

The DNA of all samples was extracted by polystyrene divinylbenzene resin (chelex) method, and the DNA of the samples was amplified by composite PCR using human fluorescent labeled STR compound amplification detection reagent. 9947A provided by the kit was used for positive control, and pure water was used to replace the DNA of the samples for negative control. Electrophoretic typing of the amplified products was determined by 3500 genetic analyzer. The genotyping results were analyzed by GeneMapper ID-X 1.5 software.

The 21 STR loci involved in this study are: D3S1358, D13S317, D7S820, D16S539, Penta E, D2S441, TPOX, TH01, D2S1338, CSF1PO, Penta D, D10S1248, D19S433, vWA, D21S11, D18S51, D6S1043, D8S1179, D5S818, D12S391, and FGA locus.

### 2.4 Statistical analysis

The two individual genotypes were compared, and the coefficient *Φ* was used to represent the probability of occurrence of homologous genes between the two individual genotypes. *Φ*_0_, *Φ*_1_, and *Φ*_2_ represent the probabilities of “no, there is one, and there are two” homologous genes, respectively, whose values satisfy the following relation hypothesis: (1) 0 ≤ *Φ*_0_, *Φ*_1_, *Φ*_2_ ≤ 1; (2) *Φ*_0_ + *Φ*_1_ + *Φ*_2_ = 1; and (3) *Φ*_1_^2^ ≥ 4*Φ*_0_ *Φ*_2_. If there is only one homologous gene between father and son, it is impossible to appear “no or there are two homologous genes,” so *Φ*_1_ = 1, *Φ*_0_ = 0 = *Φ*_2_. For example, the probability of “no, or there is 1, or there is 2” homologous genes between siblings is 1/4, 1/2, 1/4, so *Φ*_1_ = 1/2 and *Φ*_0_ = 1/4 = *Φ*_2_. Another example is two UIs, their genotypes can be the same, but the same genes are not caused by genetics, but random, they absolutely do not have homologous genes, so *Φ*_0_ = 1 and Φ_1_ = 0 = *Φ*_2_. According to the consanguineous relationship among the members of the family, three kinds of *Φ* values among the members of different relations of the family can be inferred (Table 1).

**Table 1:** The probability that common consanguineous relationships have homologous genes (*Φ*)

Kinship	Acronym	*Φ*_0_	*Φ*_1_	*Φ*_2_
Monozygotic twins	MT	0	0	1
Parent–offspring	PO	0	1	0
Full sibling	FS	1/4	1/2	1/4
Half-siblings	HS	1/2	1/2	0
First-generation cousin	1C	3/4	1/4	0
Second-generation cousin	2C	15/16	1/16	0
Unrelated individuals	UI	1	0	0

In the ITO calculation method, I, T, and O represent the probability of individual 2 developing the genotype when two individuals in a pair of samples have 2, 1, or no homologous genes [9]. The probability that common consanguineous genes have homologous genes *Φ* two individual genotypes include three cases. (1) Two individuals do not have the same gene, if *A* is *A*_*i*_ *A*_*j*_ and *B* is *A*_*k*_ *A*_*r*_, *B* cannot have a genetic homology relationship with *A*, and the probability of *B* being *A*_*k*_ *A*_*r*_ can only be the probability of *A*_*k*_ *A*_*r*_ in a random population, that is, *O* = 2*P*_*k*_ *P*_*r*_ (no homologous gene), and since *A* and *B* cannot have one or two homologous genes, *T* = 0 = *I*. (2) Two individuals have the same gene, such as *A*_*i*_ *A*_*j*_ for *A* and *A*_*i*_ *A*_*k*_ for *B*; there is no case of two homologous genes, so *I* = 0. Because they have the same gene, the same gene may be related (in this case, two people have one homologous gene), or they may not be related, and only one of the same genes appears randomly (two people have no homologous genes). Thus, the probability that *B* is *A*_*i*_ *A*_*k*_ *T* = the chance that A will produce A gene × the frequency of *A*_*k*_ gene in a random population = 0.5*P*_*k*_ (with 1 homologous gene), *O* = 2*P*_*i*_ *P*_*k*_ (without homologous gene). (3) If two individuals have two identical genes, such as *A*_*i*_ *A*_*j*_ for *A* and *A*_*i*_ *A*_*j*_ for *B*, there are three possibilities for the two identical genes: (1) both can be genetically identical (both genes are homologous genes), (2) one is genetically identical and the other is randomly identical (only one homologous gene), and (3) both are randomly identical (no homologous genes). Thus, the probability that *B* is *A*_*i*_ *A*_*j*_ is *I* = 1 (with 2 homologous genes), *T* = 0.5(*P*_*i*_ + *P*_*j*_) (with 1 homologous gene), and *O* = 2*P*_*i*_ *P*_*j*_ (without homologous genes) (Table 2). In this article, the ITO calculation method is used to calculate and count the following indices, including monozygotic twins index (MTI), parent–offsprings index (POI), full sibling index (FSI), half-sibling index (HSI), first-generation cousin index (1CI), second-generation cousin index (2CI), and unrelated individuals index (UII).

**Table 2:** Complex allele loci I, T, and O values

*A*		*B*			
		*A*_*I*_ *A*_*i*_	*A*_*i*_ *A*_*j*_	*A*_*j*_ *A*_*j*_	*A*_*j*_ *A*_*k*_
*A*_*i*_ *A*_*i*_	I	1	0	0	0
	T	*P*_*i*_	*P*_*j*_	0	0
	O	\[{P}_{i}^{2}]\]	2*P*_*i*_ *P*_*j*_	\[{P}_{j}^{2}]\]	2*P*_*j*_ *P*_*k*_

*A*		*B*						
		*A*_*i*_ *A*_*i*_	*A*_*j*_ *A*_*j*_	*A*_*i*_ *A*_*j*_	*A*_*i*_ *A*_*k*_	*A*_*j*_ *A*_*k*_	*A*_*k*_ *A*_*k*_	*A*_*k*_ *A*_*r*_
*A*_*i*_ *A*_*j*_	I	0	0	1	0	0	0	0
	T	0.5*P*_*i*_	0.5*P*_*j*_	0.5(*P*_*i*_ + *P*_*j*_)	0.5*P*_*k*_	0.5*P*_*k*_	0	0
	O	*P*_*i*_^2^	*P*_*j*_^2^	2*P*_*i*_ *P*_*j*_	2*P*_*i*_ *P*_*k*_	2*P*_*j*_ *P*_*k*_	*P*_*k*_^2^	2*P*_*k*_ *P*_*r*_

The maximum value of ITO indicators for each sample pair is calculated, and then, the classification statistics are performed according to the true kinship category of the sample pair.

## 3 Results

### 3.1 Distribution of ITO index for different kinship samples

The distribution range of ITO indexes of the MT samples (28 pairs) is shown in Table 3. The UII of all MT samples is 1, and the values of all other ITO index are more than 10. The maxima, minima, and median of the MT samples UII are the smallest, and the maxima, minima, and median of the MTI are the largest among the indexes.

**Table 3:** Distribution of ITO indexes in MT samples (*n* = 28)

Range	MTI	POI	FSI	HSI	1CI	2CI	UII
0–1	0	0	0	0	0	0	0
1–10	0	0	0	0	0	0	28
10–10^2^	0	0	0	0	0	20	0
10^2^–10^4^	0	0	0	0	0	8	0
10^4^–10^8^	0	0	0	13	28	0	0
10^8^–10^10^	0	0	0	11	0	0	0
10^10^–10^15^	0	28	0	4	0	0	0
10^15^–10^20^	0	0	23	0	0	0	0
≥10^20^	28	0	5	0	0	0	0
Maxima	1.26 × 10^32^	6.72 × 10^14^	3.09 × 10^22^	3.85 × 10^10^	1.61 × 10^7^	881.65	1.00
Minima	5.55 × 10^24^	1.55 × 10^10^	8.26 × 10^15^	4.77 × 10^6^	1.27 × 10^4^	19.40	1.00
Median	3.01 × 10^27^	1.01 × 10^12^	2.18 × 10^18^	1.24 × 10^8^	1.23 × 10^5^	47.97	1.00

Note: Range of values in the table, with the left value means ≥ and the right value means <; the numbers in the table are the number of samples for the corresponding exponential range. The following is the same.

The distribution range of ITO indexes in the PO samples (4,000 pairs) is shown in Table 4. The MTI of all PO samples is 0, the UII is 1, and all other index values are more than 1. The FSI maxima is the largest of all indexes, and the minima and median of POI are the largest of all indexes, The MTI maxima, minima, and median are the smallest of all indexes, all of which are 0.

**Table 4:** Distribution of ITO indexes in PO samples (*n* = 4,000)

Range	MTI	POI	FSI	HSI	1CI	2CI	UII
0	4,000	0	0	0	0	0	0
0–1	0	0	0	0	0	0	0
1–10^2^	0	0	74	2	255	3,813	4,000
10^2^–10^4^	0	15	874	813	3,160	177	0
10^4^–10^6^	0	610	1,715	2,534	557	1	0
10^6^–10^8^	0	2,024	1,032	607	18	0	0
≥10^8^	0	1,351	305	44	10	9	0
Maxima	0	2.09 × 10^34^	1.49 × 10^39^	2.86 × 10^29^	5.28 × 10^24^	1.32 × 10^16^	1.00
Minima	0	223.68	1.23	31.93	7.46	1.77	1.00
Median	0	2.22 × 10^7^	1.65 × 10^5^	6.88 × 10^4^	9.90 × 10^2^	9.42	1.00

The distribution range of ITO indexes of each affinity of FS (392 pairs) is shown in Table 5. The MTI of all FS samples is 0, the UII is 1, and the HSI, 1CI, and 2CI are more than 1. The POI of 36 pairs of samples is more than 0 (9.18%), and the POI of the remaining 356 pairs of samples is equal to 0. The FSI of 1 pair of samples is less than 1 (0.26%), and the FSI of other samples is more than 1. The FSI maxima and median are the largest of all indexes, and the HSI minima is the largest of all indexes. The MTI maxima, minima, and median are the smallest of all indexes, all of which are 0.

**Table 5:** Distribution of ITO indexes in FS samples (*n* = 392)

Range	MTI	POI	FSI	HSI	1CI	2CI	UII
0	392	356	0	0	0	0	0
0–1	0	0	1	0	0	0	0
1–10^2^	0	0	16	12	70	375	392
10^2^–10^4^	0	0	61	163	278	16	0
10^4^–10^6^	0	2	114	175	43	0	0
10^6^–10^8^	0	12	94	38	0	0	0
10^8^–10^10^	0	16	80	3	0	0	0
10^10^–10^15^	0	6	24	0	0	1	0
10^15^–10^20^	0	0	1	0	1	0	0
≥10^20^	0	0	1	1	0	0	0
Maxima	0	2.79 × 10^12^	1.74 × 10^29^	7.03 × 10^22^	1.22 × 10^19^	1.11 × 10^12^	1.00
Minima	0	0	0.13	6.72	6.08	1.90	1.00
Median	0	0	1.30 × 10^6^	1.52 × 10^4^	505.95	7.63	1.00

The distribution range of ITO indexes of HS (138 pairs) is shown in Table 6. In the HS samples, both the MTI and POI were 0, and the UII was both 1. In the HS samples, the ITO indexes were less than 1, with 67 pairs (48.55%) for FSI, 11 pairs (7.97%) for HSI, 6 pairs (4.35%) for 1CI, 4 pairs (2.90%) for 2CI, and all other ITO indexes more than 1. The maxima value of FSI is higher than that of the remaining indicators, the minima value of UII is the largest of all indicators, the value is 1, and the median of HSI is the largest. The maxima, minima, and median of MTI and POI are the smallest with a value of 0.

**Table 6:** Distribution of ITO indexes in HS samples (*n* = 138)

Range	MTI	POI	FSI	HSI	1CI	2CI	UII
0	138	138	0	0	0	0	0
0–10^−2^	0	0	22	1	0	0	0
10^−2^–10^−1^	0	0	21	3	1	0	0
10^−1^–1	0	0	24	7	5	4	0
1–10^2^	0	0	43	75	110	133	138
10^2^–10^4^	0	0	22	47	21	1	0
≥10^4^	0	0	6	5	1	0	0
Maxima	0	0	3.45 × 10^7^	7.56 × 10^5^	3.24 × 10^4^	181.88	1.00
Minima	0	0	1.27 × 10^−9^	1.30 × 10^−4^	0.03	0.45	1.00
Median	0	0	1.32	39.15	16.87	2.58	1.00

The distribution range of ITO indexes of UI (3,500 pairs) is shown in Table 7. All UI samples have an MTI and POI of 0 and a UII of 1. Among the UI samples, 30 pairs (0.86%) of FSI, 326 pairs (9.31%) of HSI, 859 pairs (24.54%) of 1CI, and 1,427 pairs (40.77%) of 2CI were included in the ITO index less than or equal to 1. In this article, the FSI, his, and 1CI of 1 pair of UI were observed the values are relatively high, over 10^10^ orders of magnitude. The maxima value of HSI was higher than that of the other groups, the minima and median values of each ITO index were less than or equal to 1, and the minima and median of UII were the largest of 1.

**Table 7:** Distribution of ITO indexes in UI samples (*n* = 3,500)

Range	MTI	POI	FSI	HSI	1CI	2CI	UII
0	3,500	3,500	0	0	0	0	0
0–10^−10^	0	0	4	0	0	0	0
10^−10^–10^−8^	0	0	112	0	0	0	0
10^−8^–10^−4^	0	0	2,215	21	0	0	0
10^−4^–10^−2^	0	0	950	1,079	4	0	0
10^−2^–10^−1^	0	0	140	1,236	438	0	0
10^−1^–1	0	0	49	838	2,199	2,073	0
1–10	0	0	22	272	818	1,423	3,500
10–10^2^	0	0	4	48	37	0	0
10^2^–10^4^	0	0	0	2	0	0	0
10^4^–10^8^	0	0	3	2	2	3	0
10^8^–10^10^	0	0	0	1	1	1	0
≥10^10^	0	0	1	1	1	0	0
Maxima	0	0	1.24 × 10^12^	1.90 × 10^13^	1.11 × 10^12^	1.50 × 10^8^	1
Minima	0	0	9.61 × 10^−12^	5.47 × 10^−6^	6.88 × 10^−3^	0.33	1
Median	0	0	1.71 × 10^−5^	0.04	0.42	0.92	1

### 3.2 Pairwise comparison of ITO indexes of different kinship samples

After comparing the ITO indexes of the same sample, and then summarizing the ratios of the same kinship type, it is found that the distribution of the ITO index ratios in the MT samples is shown in Table 8. The maxima, minima, and median of MTI/POI are the largest among the indicators, and the maxima, minima, and median of POI/FSI are the smallest, all less than 1.

**Table 8:** Distribution of ITO index ratios in identical in MT samples (*n* = 28)

Range	MTI/POI	POI/FSI	FSI/HSI	HSI/1CI	1CI/2CI	2CI/UII
0–10^−4^	0	28	0	0	0	0
10^−4^–10^−2^	0	0	0	0	0	0
10^−2^–1	0	0	0	0	0	0
1–10^2^	0	0	0	0	0	20
10^2^–10^4^	0	0	0	28	23	8
10^4^–10^6^	0	0	0	0	5	0
≥10^6^	28	0	28	0	0	0
Maxima	1.87 × 10^17^	3.54 × 10^−6^	8.03 × 10^11^	2.40 × 10^3^	1.82 × 10^4^	881.65
Minima	1.91 × 10^14^	2.17 × 10^−8^	1.27 × 10^9^	3.75 × 10^2^	6.22 × 10^2^	19.40
Median	2.40 × 10^15^	5.56 × 10^−7^	1.13 × 10^10^	8.79 × 10^2^	2.47 × 10^3^	47.97

Table 9 shows the distribution of the ratio of each ITO index in the PO sample. The maxima value of 2CI/UII is the largest, the minima value of HSI/1CI is the largest of all indicators, and the median of POI/FSI is the largest of all indicators. The maxima, minima, and median of MTI/POI are the smallest, all of which are 0.

**Table 9:** Distribution of ITO index ratios in identical in PO samples (*n* = 4,000)

Range	MTI/POI	POI/FSI	FSI/HSI	HSI/1CI	1CI/2CI	2CI/UII
0–10^−5^	4,000	0	0	0	0	0
10^−5^–10^−2^	0	3	1	0	0	0
10^−2^–1	0	46	1,500	0	0	0
1–10^2^	0	1,667	2,269	2,827	2,000	3,813
10^2^–10^4^	0	2,265	221	1,166	1,991	177
10^4^–10^6^	0	19	6	7	1	1
10^6^–10^8^	0	0	0	0	5	0
≥10^8^	0	0	3	0	3	9
Maxima	0	3.0 × 10^4^	5.2 × 10^9^	9.4 × 10^4^	5.9 × 10^8^	1.3 × 10^16^
Minima	0	1.41 × 10^−5^	9.93 × 10^−3^	4.28	4.20	1.77
Median	0	144.53	2.08	67.70	100.00	9.42

The distribution of ITO index ratios for FS samples is shown in Table 10. The maximum value of 2CI/UII is the largest, the minimum value of 1CI/2CI is the largest of the indicators, and the median of FSI/HSI is the largest of the indicators. The maxima, minima, and median of MTI/POI are the smallest; all are 0 and, in addition, the minima and median of POI/FSI are also 0.

**Table 10:** Distribution of ITO index ratios in identical in FS samples (*n* = 392)

Range	MTI/POI	POI/FSI	FSI/HSI	HSI/1CI	1CI/2CI	2CI/UII
0	392	356	0	0	0	0
0–1	0	20	32	0	0	0
1–10^2^	0	16	170	344	258	375
10^2^–10^4^	0	0	157	48	133	16
10^4^–10^6^	0	0	31	0	0	0
10^6^–10^8^	0	0	2	0	1	0
≥10^8^	0	0	0	0	0	1
Maxima	0	77.19	1.15 × 10^7^	5.78 × 10^3^	1.10 × 10^7^	1.11 × 10^12^
Minima	0	0	0.02	1.11	3.20	1.90
Median	0	0	83.79	27.85	59.48	7.63

The distribution of ITO index ratios for HS samples is shown in Table 11. MTI/POI is meaningless due to the denominator of 0, the maximum value of FSI/HSI is the largest of all indicators, the minimum value of 2CI/UII is the largest, and the median of 1CI/2CI is the largest. The maxima, minima, and median of POI/FSI are the smallest, all of which are 0.

**Table 11:** Distribution of ITO index ratios in identical in HS samples (*n* = 138)

Range	MTI/POI	POI/FSI	FSI/HSI	HSI/1CI	1CI/2CI	2CI/UII
0	—	138	0	0	0	0
0–10^−2^	—	0	44	1	0	0
10^−2^–10^−1^	—	0	51	1	1	0
10^−1^–1	—	0	34	35	6	4
1–10^2^	—	0	8	101	128	133
10^2^–10^4^	—	0	1	0	3	1
≥10^4^	—	0	0	0	0	0
Maxima	—	0	3.91 × 10^3^	66.68	196.45	181.88
Minima	—	0	9.79 × 10^−6^	4.78 × 10^−3^	0.06	0.45
Median	—	0	0.03	2.11	6.39	2.58

The distribution of ITO index ratios for UI samples is shown in Table 12. MTI/POI is meaningless because the denominator is 0, and the minimum values of the rest of the indicators are 0. The FSI/HSI maxima is the largest of all indicators, the median of 1CI/2CI is the largest, the maxima and median of POI/FSI is the smallest at 0, and the median of FSI/HSI is also 0.

**Table 12:** Distribution of ITO index ratios in identical in UI samples (*n* = 3,500)

Range	MTI/POI	POI/FSI	FSI/HSI	HSI/1CI	1CI/2CI	2CI/UII
0	—	138	0	0	0	0
0–10^−2^	—	0	44	1	0	0
10^−2^–10^−1^	—	0	51	1	1	0
10^−1^–1	—	0	34	35	6	4
1–10^2^	—	0	8	101	128	133
10^2^–10^4^	—	0	1	0	3	1
≥10^4^	—	0	0	0	0	0
Maxima	—	0	3,909	67	196	182
Minima	—	0	0	0	0	0
Median	—	0	0	2	6	3

### 3.3 Maximum ITO index for samples of different relatives

The ITO indices of the same sample are compared within a sample, the maximum value is collected, and the sample number, maxima, minima value, and median of the ITO maximum item are counted. The maximum ITO item of the MT sample is MTI, which accounts for up to 100%, as shown in Table 13.

**Table 13:** Maximum distribution of ITO index within MT samples

ITO indicators	Number of sample pairs	Percentage	Maxima	Minima	Median
MTI	28	100	1.26 × 10^32^	5.55 × 10^24^	3.01 × 10^27^

The maximum distribution of ITO index for PO samples is shown in Table 14. The maximum ITO items of PO samples include POI and FSI, and the proportion of POI is much higher than that of FSI, as high as 98.78%, but the maxima, minima, and median of POI are smaller than FSI.

**Table 14:** Maximum distribution of ITO index within PO samples

ITO indicators	Number of sample pairs	Percentage	Maxima	Minima	Median
POI	3,951	98.78	1.29 × 10^32^	223.68	2.19 × 10^7^
FSI	49	1.23	1.49 × 10^39^	3.44 × 10^4^	4.51 × 10^8^

The maximum distribution of ITO index for the FS sample is shown in Table 15. The maximum ITO items of the FS sample include POI, FSI, and HSI, of which FSI is the highest proportion, 87.76%, of which the maximum value of FSI is the largest of the three.

**Table 15:** Maximum distribution of ITO index within FS samples

ITO indicators	Number of sample pairs	Percentage	Maxima	Minima	Median
POI	16	4.08	1.22 × 10^9^	2.15 × 10^5^	4.86 × 10^7^
FSI	344	87.76	1.74 × 10^29^	32.06	3.19 × 10^6^
HSI	32	8.16	1.03 × 10^5^	6.72	347.00

The maximum distribution of ITO index for HS samples is shown in Table 16. The maximum ITO items of the HS sample include FSI, HSI, 1CI, 2CI, and UII, of which HSI is the highest proportion, 66.67%, of which the maximum, minimum, and median of FSI are the largest among all indicators.

**Table 16:** Maximum distribution of ITO index within HS samples

ITO indicators	Number of sample pairs	Percentage	Maxima	Minima	Median
FSI	9	6.52	3.45 × 10^7^	178.32	7.89 × 10^2^
HIS	92	66.67	7.56 × 10^5^	4.89	82.93
1CI	30	21.74	13.91	1.33	3.54
2CI	3	2.17	1.24	1.14	1.23
UII	4	2.90	—	—	1

The maximum distribution of ITO index for UI samples is shown in Table 17. The ITO maximum items of the UI sample include HSI, 1CI, 2CI, and UII, of which UII is the highest proportion, 59.23%, and the maxima, minima, and median of HSI are the largest of all indicators.

**Table 17:** Maximum distribution of ITO index within UI samples

ITO indicators	Number of sample pairs	Percentage	Maxima	Minima	Median
HS	90	2.57	1.90 × 10^13^	3.03	10.49
1C	573	16.37	2.56 × 10^6^	1.10	2.04
2C	764	21.83	2.93	1.00	1.12
UR	2,073	59.23	1.00	1.00	1.00

For two sample individuals that are unknown, it is a common method to judge their true kinship from the perspective of the ITO index maximum term. In this study, Bayes’ theorem is used to determine the probability that the maximum term of the ITO index is a certain affinity type (assuming an equal chance of the kinship types that make up a complete group of events), as shown in Table 18. Except for the maximum ITO index item of the MT sample, which only includes MTI, the maximum value of the ITO index of the remaining four groups of samples contains multiple indicators, but the maximum value of the ITO index with the largest proportion of each group of samples is consistent with the sample type.

**Table 18:** Distribution of ITO index maximums in actual kinship types (pairs, %)

item	MT		PO		FS		HS		UI	
	Amount	Proportion (%)	Amount	Proportion (%)	Amount	Proportion (%)	Amount	Proportion (%)	Amount	Proportion (%)
MTI	28	100								
POI			3,951	98.78	16	4.08				
FSI			49	1.23	344	87.76	9	6.52		
HSI					32	8.16	92	66.67	90	2.57
1CI							30	21.74	573	16.37
2CI							3	2.17	764	21.83
UII							4	2.90	2,073	59.23
Total	28	100	4,000	100	392	100	138	100	3,500	100

## 4 Discussion

In the identification of all siblings to UIs, multiple STR loci were jointly detected, and the number of all identical and completely different loci could effectively indicate the sibling relationship [10]. The separate ITO index statistics involved in this study show that the MTI and UII distributions are relatively single, that is, all UIIs are 1, and the MTI of all samples except the MT sample is 0. However, the four indicators of FSI, HSI, 1CI, and 2CI are relatively complex, with phenomena greater than 1 and phenomena less than 1. Observing the median distribution of ITO index, it can be found that from MT, PO, FS, HS to UI, the more distant the kinship, the smaller the ITO index. To a certain extent, the median indicates that there is a relationship between STR locus matching and sibling relationship, and it is necessary to further determine the cut-off value to determine the relationship between each sample to effectively reduce the prediction error [11].

In the process of calculating the interbody kinship relationship of the ITO algorithm, the comparison of the ITO index of the same sample is a common effective index [12]. In this study, even if all index items such as MTI or POI are not considered, there are cases where the ratio of some indices is greater than 1 and the ratio of other indices is less than 1. That is to say, from the MTI to UII direction, the ITO index values of the same sample are not always simply arranged from high to low. Studies have shown that when the ITO method identifies FS and HS, FSI/HSI ≥ 1 can be used as the FS discrimination standard, and FSI/HSI < 1 can be used as the HS discrimination standard, with an accuracy rate of 87.5%, which is similar to the results of this study [13]. If the MTI is not 0, the sample type can be determined to be MT. When the maximum and minimum values of POI are not 0, the sample can be judged to be PO. When the maximum POI is not 0 and the minimum value is 0, the sample can be identified as FS. In addition, this study found that the MTI/POI of the MT sample was the largest, the median POI/FSI of the PO sample was the largest, and the median of 1CI/2CI of the UI sample was the largest, which may be the criterion for distinguishing MT, PO, and UI. However, the critical point of ITO index of different kinships is not clear, there is a certain error, and further research is still needed.

In all the samples in this article, most of the maximum ITO index values (>50%) of the same type of sample appeared in the ITO index item where their true kinship is located, and the specific proportion varied with the type of kinship. The specific proportion was 100% (MTI) for MT samples and 98.78% (POI) for PO samples. 87.76% (FSI) of the FS sample; The HS sample was 66.67% (HSI); The sample of UI was 59.23% (UII). According to the distance of kinship, from the direction of MT, PO, FS, HS, and UI, the proportion of the maximum ITO index of the same type of sample in the true kinship index item showed a decreasing trend.

This study shows that the maximum term of ITO index as the basis for judging the kinship of two bodies is a noteworthy idea. For example, when the maximum value of the ITO index is FSI, the true kinship is determined to be FS; When the maximum value of the ITO index is HSI, the true relative is determined to be HS. Based on this idea, the accuracy of judging the kinship of the sample is judged, and the accuracy rate is as follows: MT 100%, PO 96.03%, FS 91.89%, HS 86.13%, and UI 95.33%.

ITO index has more autosomal STR detection, and the results show that the number of STR tests will affect the judgment of results [14], and ITO is also used more in FS and HS sample discrimination, while the application of ITO index in multiple sample discrimination has not been reported [15]. In this study, not only more autosomal STRs were detected, but also ITO index analysis was carried out together with a variety of sample types, which expanded the application of ITO index in practical applications and increased the scope of ITO identification to confirm all kinship. At the same time, the author finds that taking the maximum term of ITO index as the basis for judging the kinship of two bodies has good application value. When the sample affinity is MT, PO, and FS, the TOI index can not only accurately identify the kinship but also have the advantages of simple and fast calculation.

However, our study had some limitations, as the sample included in this article did not involve first-generation cousin (1C) and second-generation cousin (2C), so the effects of these two types were not considered here. At the same time, the determination of the discriminant cut-off values of different samples still needs further research.

## 5 Conclusion

MTI is not 0 to identify relatives as MT, POI is an important indicator to distinguish between relatives as PO and FS. The critical values of ITO index discriminant values for UI and HS need to be further studied.
